
JOURNAL INFORMATION
==============================
NLM Title Abbreviation: Wirtschaftsdienst
Journal ID: Wirtschaftsdienst
NLM Journal ID: 101086612

Wirtschaftsdienst (Hamburg, Germany : 1949)

ISSN: 0043-6275

ARTICLE INFORMATION
==============================
PMCID: PMC8857622
PMID: 35221387
DOI: 10.1007/s10273-022-3109-4
Article ID: 3109
Article version: 1
Subjects: Zeitgespräch

Beim Übergang zum Bürgergeld mutig große Veränderungen wagen

Introducing the Citizen’s Income (Bürgergeld): Making Bold Changes

Herzog-Stein Alexander alexander-herzog-stein@boeckler.de

Ref. Makroökonomische Arbeitsmarktforschung, Institut für Makroökonomie u. Konjunkturforschung, Hans-Böckler-Straße 39, 40476 Düsseldorf, Deutschland
Electronic publication date: 2022 Feb 19
Print publication date: 2022
Volume: 102
Issue: 2
First page: 100
Last page: 103
Copyright: © Der/die Autor 2022
License: Dieser Artikel wird unter der Creative Commons Namensnennung 4.0 International Lizenz veröffentlicht (creativecommons.org/licenses/by/4.0/deed.de).
Open Access wird durch die ZBW — Leibniz-Informationszentrum Wirtschaft gefördert.
License URL: https://creativecommons.org/licenses/by/4.0/

Keywords: J65, J68
issue-copyright-statement: © The Author(s) 2022

==============================
The new German federal government wants to replace the unemployment benefit II (Arbeitslosengeld II) with a so-called citizen’s income (“Bürgergeld”). In doing so, it needs to make major changes. Measures outlined in the coalition agreement indicate that the existing imbalance in the so called support and claim-approach (“Fördern und Fordern”) for the long-term unemployed will be finally readjusted towards supportive policy measures such as training. The reform of the in-work benefit element should aim at supporting sustainable employment subject to social security contributions. However, to live up to its name, the citizen’s income must ensure genuine social participation for those receiving the citizen’s income.

Prof. Alexander Herzog-Stein, Ph. D., ist Leiter des Referats Arbeitsmarktökonomik am Institut für Makroökonomie und Konjunkturforschung (IMK) in Düsseldorf und Honorarprofessor an der Universität Koblenz-Landau.

Mit dem Ende 2003 verabschiedeten vierten Gesetz für moderne Dienstleistungen am Arbeitsmarkt (Hartz IV) fanden die Arbeitsmarktreformen der damaligen rot-grünen Bundesregierung ihren bis heute umstrittenen Abschluss. Das bis dahin bestehende Versicherungs- und Transfersystem im Falle von Arbeitslosigkeit wurde grundlegend verändert. Die am letzten Arbeitsgehalt orientierte Arbeitslosenhilfe und die bedarfsorientierte Sozialhilfe wurden durch die bedarfsorientierte Grundsicherung für Arbeitsuchende (Arbeitslosengeld II) ersetzt. Die Höhe des Arbeitslosengelds II entsprach in etwa dem Leistungsniveau der bisherigen Sozialhilfe. Im Vergleich zur Arbeits- und Sozialhilfe wurde zudem ein umfassendes arbeitnehmerseitiges Kombilohnelement durch die nur teilweise Anrechnung von Erwerbseinkommen geschaffen (SVR, 2006). Zusätzlich wurde mit dem ebenfalls Ende 2003 verabschiedeten Gesetz zu Reformen am Arbeitsmarkt die Bezugsdauer des Arbeitslosengelds im Rahmen der Arbeitslosenversicherung stark verkürzt.

Obwohl die Hartz-IV-Reform bis heute umstritten ist und wiederholt von wissenschaftlicher und politischer Seite Reformen am Arbeitslosengeld II gefordert wurden[1], hat sich nunmehr mit der neuen Koalitionsregierung aus SPD, Bündnis 90/Die Grünen und FDP erstmals eine Bundesregierung für eine grundsätzliche Reform des Arbeitslosengelds II entschieden: Sie beabsichtigt es durch ein neues Bürgergeld zu ersetzen. Anhand des Koalitionsvertrags (SPD et al., 2021) setzt sich dieser Beitrag kritisch mit diesem Vorhaben auseinander.

## Eine kurze Bestandsaufnahme der Hartz-IV-Reform

Die Hartz-Reformen und das Arbeitslosengeld II waren Kinder ihrer Zeit. Mit der Einführung des Bürgergelds müssen deshalb die Prämissen des Arbeitslosengelds II kritisch hinterfragt werden. Die Erfahrungen und Erkenntnisse der letzten 17 Jahre können bei der Ausgestaltung des Bürgergelds helfen. Laut Bäcker (2019) war für das Arbeitslosengeld II eine Sichtweise handlungsleitend, die die Ursachen der hohen Arbeitslosigkeit nicht mehr auf gesamtwirtschaftliche Ungleichgewichte zwischen Angebot und Nachfrage, sondern vor allem „… auf die Eigenschaften, Fähigkeiten und Verhaltensweisen der Arbeitslosen“ (S. 253) zurückführte. Entsprechend setzte man auf eine Angebotspolitik, die darauf abzielte, die Unternehmenskosten zu reduzieren und den Betrieben Anreize zu geben, mehr Beschäftigung zu schaffen. Ziel der Arbeitsmarktreformen war es insbesondere, die Löhne für Geringverdienende zu senken und so den Niedriglohnsektor auszuweiten (Herzog-Stein et al., 2013).

Nun, 17 Jahre später, wissen wir mehr oder doch nicht? Von 2005 bis 2019 – dem letzten Jahr vor Ausbruch der COVID-19-Pandemie – ging die registrierte Arbeitslosigkeit in Deutschland um insgesamt 6,7 Prozentpunkte zurück. Wie groß der Beitrag der Hartz-IV-Reform am Rückgang der Arbeitslosigkeit war, ist umstritten.

Die Ergebnisse der wissenschaftlichen Literatur zur Wirkung der Hartz-IV-Reform, die vor allem aus makroökonomischen Simulationsanalysen besteht, sind ernüchternd. Krebs (2019) schätzt auf der Grundlage seiner eigenen Analysen und den Ergebnissen anderer Untersuchungen den Beitrag der Hartz-IV-Reform zur Reduktion der Arbeitslosigkeit in Deutschland auf höchstens einen Prozentpunkt. Bradley und Kügler (2019) untersuchen die Wirkung aller Hartz-Reformen und kommen zu dem Schluss, dass sie kaum das Beschäftigungsniveau beeinflusst haben; gleichzeitig senkte die Hartz-IV-Reform aber die Löhne merklich.

Bei der Neugestaltung des Bürgergelds sollten die Erfahrungen der vergangenen Jahre berücksichtigt werden. Auch die Beschäftigungseffekte des Bürgergelds

1 Siehe exemplarisch das Zeitgespräch „Hartz IV – Reform einer umstrittenen politischen Maßnahme“ im Heft 4/2019 dieser Zeitschrift, https://www.wirtschaftsdienst.eu/inhalt/jahr/2019/heft/4/beitrag/hartz-iv-reform-einer-umstrittenen-politischen-massnahme.html (2. Februar 2022).

**Prof. Alexander Herzog-Stein, Ph. D.**, ist Leiter des Referats Arbeitsmarktökonomik am Institut für Makroökonomie und Konjunkturforschung (IMK) in Düsseldorf und Honorarprofessor an der Universität Koblenz-Landau.

dürften überschaubar bleiben. Eine gute wirtschaftliche Entwicklung mit einer dynamischen Entwicklung der Binnennachfrage hingegen schafft neue gute Arbeitsplätze und reduziert die Arbeitslosigkeit nachhaltig. Eine aktive Wirtschafts- und Fiskalpolitik, die in die Zukunft investiert, leistet hierzu einen wesentlichen Beitrag. In einem positiven wirtschaftlichen Umfeld, in dem viele neue Arbeitsplätze entstehen, kann dann ein effektiv ausgestaltetes Bürgergeld die Situation von Arbeitslosen und ihre Arbeitsmarktchancen verbessern.

### Gleichgewicht zwischen Fördern und Fordern herstellen

Die notwendige sozialökologische Transformation als Antwort auf die Herausforderungen des Klimawandels und die Auswirkungen des demografischen Wandels auf dem Arbeitsmarkt verlangen in den nächsten Jahren große Anstrengungen und bringen Veränderungen mit sich. Verstärkt muss es deshalb darum gehen, ungenutzte Arbeitsmarktpotenziale zu heben, insbesondere unter den arbeitslosen Erwerbspersonen. Im Jahresdurchschnitt 2021 waren immer noch 2,61 Mio. Personen registriert arbeitslos; davon befanden sich 61,8 % in der Grundsicherung für Arbeitssuchende. Arbeitslose Erwerbspersonen, insbesondere aus dem Bereich des SGB II, möglichst über dauerhafte sozialversicherungspflichtige Beschäftigung wieder nachhaltig in den Arbeitsmarkt zu integrieren, verlangt besondere Anstrengungen. Bildung und Qualifikation sind hierbei in der sich verändernden Arbeitswelt von zentraler Bedeutung.

Deshalb muss mit dem Bürgergeld endlich die seit Einführung des Arbeitslosengelds II bestehende Unwucht zulasten des Förderns behoben werden. Durch eine stärkere Betonung des Förderns und eine umfassende und zielgenaue Betreuung im Bürgergeld kann es gelingen, die qualifikatorischen Voraussetzungen zu schaffen, dass arbeitslose Bürgergeldbeziehende, die häufig multiple Vermittlungshemmnisse aufweisen, in der neuen Arbeitswelt besser bestehen können. Der Fokus auf niedrigqualifizierte und schlecht bezahlte Tätigkeiten, der in der Vergangenheit die Arbeitswelt der Grundsicherung prägte, ist nicht mehr zeitgemäß und würde für die Zukunft die Gefahr eines verstärkten qualifikatorischen Mismatch zwischen Arbeitsangebot und -nachfrage mit sich bringen.

Es ist deshalb zu begrüßen, dass die Bundesregierung den Vermittlungsvorrang nach § 3 Abs. 1 Satz 3 SGB II beim Bürgergeld abschaffen will. Die angestrebte Stärkung der Förderung von Weiterbildung, insbesondere auch von vollqualifizierenden Ausbildungen, und der Qualifizierung der Arbeitssuchenden sowie verbesserte Betreuungsschlüssel im Bürgergeld sind richtig, um ein Gleichgewicht zwischen dem Fördern und Fordern herzustellen. Dazu passt die Absicht, Arbeitslose bei beruflicher Qualifizierung künftig durch ein zusätzliches Weiterbildungsgeld in Höhe von 150 Euro pro Monat finanziell besserzustellen und ihnen nach Ende der Weiterbildung einen Arbeitslosengeldanspruch von mindestens drei Monaten zu gewähren. Die Politik muss sich aber bewusst sein, dass Qualifizierung und Weiterbildung einen langen Atem verlangen, da sie insbesondere mittel- und langfristig ihre positiven ökonomischen Effekte offenbaren.

### Das Kombilohnelement neu ausrichten

Die Bundesregierung plant, das bestehende arbeitnehmerseitige Kombilohnelement weiterzuentwickeln und mit anderen steuerfinanzierten Sozialleistungen abzustimmen, sodass „… die Transferentzugsraten die günstigsten Wirkungen hinsichtlich Beschäftigungseffekten und Arbeitsmarktpartizipation in sozialversicherungspflichtiger Beschäftigung erzielen, die Zuverdienstmöglichkeiten verbessert und Grenzbelastungen von 100 und mehr Prozent ausgeschlossen werden“ (SPD et al., 2021, 77).

Dies ist generell zu begrüßen, denn im Arbeitslosengeld II ist die Zielsetzung des arbeitnehmerseitigen Kombilohnelements immer unklar geblieben. Zudem sind die Effekte auf das extensive und das intensive Arbeitsangebot, die vom derzeitigen Verlauf der Transferentzugsraten ausgehen, problematisch. Die großzügigste Förderung erfolgt im Eingangsbereich des Kombilohns, denn die ersten 100 Euro Erwerbseinkommen sind anrechnungsfrei, sodass Minijobs mit einem sehr geringen Stundenvolumen finanziell attraktiv sind. Danach sind die Transferentzugsraten mit 80 % bis 100 % sehr hoch, sodass das Nettoeinkommen bei zusätzlichem Arbeitseinkommen nur sehr wenig oder gar nicht steigt.

Allerdings sollte die Bundesregierung die Transferentzugsraten des arbeitnehmerseitigen Kombilohnelements im Bürgergeld so ausrichten, dass insbesondere die Aufnahme einer sozialversicherungspflichtigen Beschäftigung von einem wöchentlichen Arbeitsumfang von mindestens 15 Arbeitsstunden gefördert und so die Arbeitslosigkeit beendet wird.[2] Es ist hierfür sinnvoll, den Verlauf der Transferentzugsraten zukünftig am Mindestlohn auszurichten und so zu gestalten, dass sie – bei Zugrundelegung eines Mindestlohns von 12 Euro – bis zu einem monatlichen Bruttoarbeitseinkommen von 783

2 Nach § 138 SGB III „Arbeitslosigkeit“ gilt eine arbeitslos gemeldete Person erst ab einer Erwerbstätigkeit mit einem wöchentlichen Arbeitsvolumen von mindestens 15 Stunden als nicht mehr arbeitslos.

Euro (= 12 Euro × 15 Arbeitsstunden × 4,35 Wochen) bei 100 % liegt. Ab dieser Verdienstgrenze sollte der Verlauf zudem möglichst attraktiv ausgestaltet sein.[3] Ziel sollte eine nachhaltige Integration in den Arbeitsmarkt sein, die durch einen Minijob mit geringem Stundenumfang nicht erzielt werden kann.

Eine solche Neuausrichtung wäre zudem ein klares Signal an die Arbeitgebendenseite; substanzielle Arbeitsnachfrageeffekte wären zu erwarten. Die bisherigen Transferentzugsraten sind eine Einladung an Betriebe, Bezieher:innen von Arbeitslosengeld II lediglich Minijobs mit einem sehr geringen Stundenvolumen anzubieten. Dies wäre zukünftig nicht mehr attraktiv und der Fokus des Kombilohnelements im Bürgergeld würde geschärft.

Im Arbeitslosengeld II berechtigte Einwände, dass für viele Haushalte diese Minijobs unverzichtbar sind, um die zu niedrigen Regelsätze des Arbeitslosengelds II etwas aufzustocken, lassen sich beim Bürgergeld prinzipiell einfach beheben. Die Regelsätze sollten endlich so berechnet werden, dass die gesellschaftliche Teilhabe der Bürgergeldbeziehenden wirklich garantiert ist. Entsprechende Vorschläge liegen vor (siehe beispielsweise Becker, 2021).

Bestimmten Personengruppen, wie beispielsweise Alleinerziehenden, denen aufgrund ihrer persönlichen oder familiären Situation ein wöchentliches Arbeitsvolumen von mindestens 15 Arbeitsstunden schwerer fallen dürfte, sollte durch konkrete Betreuungsangebote und – wo sinnvoll auch durch zusätzliche (zeitlich befristete) arbeitgeberseitige Lohnsubventionen geholfen werden. Denn für diese Personengruppen ersetzen Minijobs ebenfalls keine richtige Arbeitsmarktintegration mittels einer sozialversicherungspflichtigen Beschäftigung.

Richtig ist, dass zukünftig im Rahmen des Bürgergelds in den ersten zwei Jahren keine Vermögensanrechnung erfolgen soll, das Schonvermögen erhöht sowie die Angemessenheit der Wohnung geachtet wird. Im Hinblick auf sozialversicherungspflichtige Erwerbspersonen, deren Arbeitsvolumen 15 Wochenstunden und mehr beträgt, könnte zudem darüber nachgedacht werden, auf eine Vermögensprüfung oder -anrechnung dauerhaft weitgehend zu verzichten; die Möglichkeit von Sanktionen ist für diesen Personenkreis auf jeden Fall unangebracht und würde dem Prinzip des Förderns und Forderns widersprechen: In einem kapitalistischen Wirtschaftssystem, in dem unfreiwillige Arbeitslosigkeit ein gesamtwirtschaftliches Massenphänomen ist, haben Bürger:innen, die einer Erwerbstätigkeit nachgehen und nicht arbeitslos sind, ihren Beitrag geleistet und können vonseiten der Gesellschaft Unterstützung ohne Schikanen und Stigmatisierungen erwarten. In diesem Zusammenhang ist es sinnvoll, wie von den Koalitionsparteien angedacht, diese Personen zukünftig in der Arbeitslosenversicherung durch die Agenturen für Arbeit zu betreuen; sie könnten auch die Auszahlung des Bürgergelds an diese Personen übernehmen.

3 So haben beispielsweise Bündnis 90/Die Grünen und die FDP sowie das Institut für Arbeitsmarkt- und Berufsforschung (IAB) und das ifo-Institut Vorschläge zur Reform des Arbeitslosengelds II und damit der Transferentzugsraten veröffentlicht. Für eine tabellarische Gegenüberstellung der Vorschläge siehe Blömer et al. (2021, Tab. 1).

## Die Arbeitslosenversicherung stärken

Neben den fehlenden Aussagen zur angemessenen Höhe des neuen Bürgergelds, die notwendig wären, um das explizit genannte Ziel der Befähigung zur gesellschaftlichen Teilhabe sicherzustellen, wird leider im Koalitionsvertrag die aktuell bestehende Unwucht zwischen Arbeitslosenversicherung und Bürgergeld nicht angesprochen. Derzeit erhält der größte Teil der Arbeitslosen nicht die Versicherungsleistung Arbeitslosengeld, sondern Arbeitslosengeld II. Die Arbeitslosenversicherung als Pflichtversicherung gegen das Risiko Arbeitslosigkeit sollte jedoch wieder den Vorrang erhalten und das Bürgergeld im Falle von Arbeitslosigkeit quantitativ nachrangig sein.

Empfehlenswert wäre es deshalb, die Schutzwirkung der Arbeitslosenversicherung für Erwerbspersonen, die Beitragszahlungen geleistet haben, wieder zu erhöhen. Dies könnte durch eine Verlängerung der Bezugsdauer des Arbeitslosengelds und niedrigere Zugangshürden zum Arbeitslosengeld für Erwerbstätige in kurzfristiger und unstetiger Beschäftigung erreicht werden (für konkrete Vorschläge siehe unter anderem Bäcker, 2021).

Im Einklang mit der beabsichtigten verstärkten Förderung von Qualifizierung und Weiterbildung sollte zudem über eine Weiterentwicklung der Arbeitslosenversicherung hin zu einer Arbeitsversicherung nachgedacht werden (Schmid, 2008; Hans et al., 2017).

## Schlussbemerkungen

Eine Reform des Arbeitslosengelds II ist überfällig. Es ist begrüßenswert, dass die Bundesregierung das bisherige Arbeitslosengeld II durch ein Bürgergeld ersetzen will. Auch wenn die konkrete Ausgestaltung abgewartet werden muss, sollte die Ampelkoalition dazu ermutigt werden, den großen Schritt zu wagen und ein Bürgergeld zu schaffen, welches wirklich die gesellschaftliche Teilhabe der Bezieher:innen sicherstellt und ihnen gleichzeitig eine wirkliche Unterstützung hinsichtlich einer nachhaltigen Integration in gute Arbeit bietet.



Literatur

Bäcker G Arbeitslosenversicherung stärken! Sozialgesetzbuch III und II harmonisieren! Wirtschaftsdienst 2019 99 4 252 255
Bäcker, G. (2021), Sozialversicherung und Grundsicherung im Spannungsverhältnis — Umbrüche und Perspektiven des Systems der sozialen Sicherung, in F. Blank, C. Schäfer, D. Spannagel (Hrsg.), Grundsicherung weiterdenken, 37–60, transcript Verlag.
Becker, I. (2021), Sicherung des Existenzminimums mit Regelleistungen — Kritische Anmerkungen und Reformüberlegungen zu Hartz IV und zum Familienlastenausgleich, in F. Blank, C. Schäfer, D. Spannagel (Hrsg.), Grundsicherung weiterdenken, 61–84, transcript Verlag.
Blömer M Fuest C Peichl A Die Hartz-IV-Reformdebatte ifo-Schnelldienst 2021 72 6 21 25
Bradley J Kügler A Labor market reforms: An evaluation of the Hartz policies in Germany European Economic Review 2019 113 108 135 10.1016/j.euroecorev.2018.12.008
Hans, J. P., S. Hofmann, W. Sesselmeier und A. Yollu-Tok (2017), Umsetzung, Kosten und Wirkungen einer Arbeitsversicherung, gute gesellschaft — soziale demokratie # 2017 plus, Friedrich-Ebert-Stiftung.
Herzog-Stein, A., F. Lindner und R. Zwiener (2013), Nur das Angebot zählt — Wie eine einseitige deutsche Wirtschaftspolitik Chancen vergeben hat und Europa schadet, IMK Report, 87, Institut für Makroökonomie und Konjunkturforschung.
Krebs (2019), Welche Auswirkungen die Hartz IV-Reform auf den Rückgang der Arbeitslosigkeit hatte, https://makronom.de/welche-auswirkungen-die-hartz-iv-reform-auf-den-rueckgang-der-arbeitslosigkeit-hatte-29949 (2. Februar 2022).
Schmid, G. (2008), Von der Arbeitslosen- zur Beschäftigungsversicherung: Wege zu einer neuen Balance individueller Verantwortung und Solidarität durch eine lebenslauforientierte Arbeitsmarktpolitik, WISO Diskurs, Friedrich-Ebert-Stiftung.
SPD, Bündnis 90/Die Grünen, FDP (2021), Mehr Fortschritt Bündnis für Freiheit, Gerechtigkeit und Nachhaltigkeit. Koalitionsvertrag zwischen SPD, Bündnis 90/Die Grünen und FDP, https://www.bundesregierung.de/resource/blob/974430/1990812/a4ceb7591c8d9058b402f0a655f7305b/2021-12-10-koav2021-data.pdf (2. Februar 2022).
SVR — Sachverständigenrat zur Begutachtung der gesamtwirtschaftlichen Entwicklung (2006), Arbeitslosengeld II reformieren: Ein zielgerichtetes Kombilohnmodell, Expertise im Auftrag des Bundesministers für Wirtschaft und Technologie, Eigenverlag.
